# Supplementary material for: End Sequence Analysis Toolkit (ESAT) expands the extractable information from single-cell RNA-seq data
Source: Genome Res. 2016 Oct;26(10):1397–410. doi: 10.1101/gr.207902.116 (PMC5052061; doi:10.1101/gr.207902.116)
Supplement: Supplemental Material [file supp_gr.207902.116_Supp_Materials.tar.gz › ESAT-esat_v0.1/doc/html/functions_func.html]

ESAT: Class Members - Functions


ESAT


- Main Page
- Related Pages
- Packages
- Classes
- Files

- Class List
- Class Index
- Class Hierarchy
- Class Members

- All
- Functions
- Variables
- Enumerator

- a
- b
- c
- d
- e
- f
- g
- h
- i
- j
- k
- l
- m
- n
- o
- p
- q
- r
- s
- t
- u
- v
- w
- z


All Classes Namespaces Files Functions Variables Enumerator Pages

### - a -

- absFold()
  : broad.core.math.Statistics
- absoluteToRelativePosition()
  : umms.core.annotation.Gene
- absoluteValue()
  : broad.core.math.Statistics
- AbstractListScore()
  : umms.core.model.score.AbstractListScore
- AbstractPairedEndAlignment()
  : umms.core.alignment.AbstractPairedEndAlignment
- AbstractWindowScore()
  : umms.core.model.score.WindowScore.AbstractWindowScore
- accept()
  : broad.core.annotation.GenomicAnnotationFilter< T extends LightweightGenomicAnnotation >
- acceptRead()
  : broad.pda.seq.rap.rna.CollectRnaSeqMetrics
- acidAt()
  : jaligner.Sequence
- add()
  : broad.core.datastructures.MatrixWithHeaders
  , broad.core.math.EmpiricalDistribution
  , jaligner.Alignment
  , umms.core.alignment.AlignmentPair
  , umms.core.annotation.AnnotationList< T extends Annotation >
- addAlignment()
  : umms.core.writers.PairedEndWriter
- addAll()
  : broad.core.math.EmpiricalDistribution
  , umms.core.annotation.AnnotationList< T extends Annotation >
- AddAllIsoforms()
  : broad.pda.gene.GeneWithIsoforms
- AddAllIsoNotIncludedforms()
  : broad.pda.gene.GeneWithIsoforms
- addAnnotation()
  : broad.core.annotation.AnnotationReader< T extends GenomicAnnotation >
- addAnnotationSet()
  : broad.core.annotation.AnnotationReader< T extends GenomicAnnotation >
- addAnnotationToGraph()
  : umms.core.scripture.OrientedChromosomeTranscriptGraph
- addAnnotationToLastTrack()
  : broad.core.annotation.AnnotationReader< T extends GenomicAnnotation >
- addAttribute()
  : broad.core.annotation.GFF
  , umms.core.annotation.Gene
- addAttributeValues()
  : broad.core.annotation.GFF
- addBlock()
  : broad.core.annotation.BasicGenomicAnnotation
  , broad.core.annotation.BED
  , broad.core.annotation.GenomicAnnotation
  , broad.core.multiplealignment.MAFAlignment
  , broad.core.sequence.SequenceRegion
- addBlocks()
  : broad.core.multiplealignment.MAFAlignment
  , umms.core.annotation.BasicAnnotation
- addBooleanArg()
  : broad.core.parser.CommandLineParser
- addBrowserLine()
  : broad.core.annotation.AnnotationReader< T extends GenomicAnnotation >
- addColumn()
  : broad.core.datastructures.MatrixWithHeaders
  , broad.core.motif.PositionWeightMatrix
- addConservedPi()
  : broad.core.siphy.PiHMM
- addContainedIsoform()
  : broad.pda.gene.GeneWithIsoforms
- addDistribution()
  : broad.core.math.EmpiricalDistribution
- addDoubleArg()
  : broad.core.parser.CommandLineParser
- addEdge()
  : broad.pda.seq.graph.ChromosomeWithBubblesJGraphT
  , broad.pda.seq.graph.Path
  , umms.core.scripture.OrientedChromosomeTranscriptGraph
- addEdgeAddingMissingVertices()
  : broad.pda.seq.graph.ChromosomeWithBubblesJGraphT
- addEdges()
  : broad.pda.seq.graph.Path
- addExtraField()
  : umms.core.annotation.Gene
- addExtraScore()
  : broad.core.annotation.BasicLightweightAnnotation
  , broad.core.annotation.LightweightGenomicAnnotation
  , broad.core.sequence.SequenceRegion
- addFilter()
  : broad.core.annotation.AnnotationReader< T extends GenomicAnnotation >
  , umms.core.annotation.AnnotationCollection< T extends Annotation >
  , umms.core.annotation.AnnotationList< T extends Annotation >
  , umms.core.model.AlignmentModel
  , umms.core.model.JCSAlignmentModel
- addFilters()
  : broad.core.annotation.AnnotationReader< T extends GenomicAnnotation >
  , umms.core.annotation.AnnotationCollection< T extends Annotation >
  , umms.core.annotation.AnnotationList< T extends Annotation >
  , umms.core.model.AlignmentModel
  , umms.core.model.JCSAlignmentModel
- addFloatArg()
  : broad.core.parser.CommandLineParser
- addIntArg()
  : broad.core.parser.CommandLineParser
- addInterval()
  : umms.core.annotation.CompoundInterval
- addIsoform()
  : broad.pda.annotation.Locus
- AddIsoform()
  : broad.pda.gene.GeneWithIsoforms
- addIsoform()
  : umms.core.annotation.Gene
- addListener()
  : broad.core.siphy.TreeScalerIO
- addMatch()
  : broad.core.motif.meme.MemeMotif
- addNewEdge()
  : broad.pda.seq.graph.ChromosomeWithBubblesJGraphT
- addPairedEnd()
  : broad.pda.seq.graph.Path
- addPairedEndEdges()
  : broad.pda.seq.graph.Path
- addPseudoCounts()
  : broad.core.motif.PositionWeightColumn
  , broad.core.motif.PositionWeightMatrix
  , broad.core.motif.PositionWeightMatrixIO
- addPsuedocounts()
  : broad.core.math.EmpiricalDistribution
- addPWMColumn()
  : broad.core.motif.meme.MemeMotif
- addRecord()
  : umms.core.writers.PairedEndWriter
- addRefSeq()
  : broad.pda.annotation.BEDFileParser
- addRefSeqSet()
  : broad.pda.annotation.BEDFileParser
- addRow()
  : broad.core.datastructures.MatrixWithHeaders
- addScore()
  : broad.pda.datastructures.Alignments
- addSequence()
  : broad.core.multiplealignment.MAFAlignment
  , broad.core.multiplealignment.MultipleAlignment
  , umms.core.annotation.Gene
- addSequences()
  : broad.core.multiplealignment.MAFAlignment
  , broad.core.multiplealignment.MultipleAlignment
- addShortEncodedColumn()
  : broad.core.multiplealignment.MAFAlignment
  , broad.core.multiplealignment.MultipleAlignment
- addShortEncodedColumnMatrix()
  : broad.core.multiplealignment.MultipleAlignment
- addShortEncodedRegion()
  : broad.core.multiplealignment.MAFAlignment
  , broad.core.multiplealignment.MultipleAlignment
- addSourceAnnotation()
  : umms.core.feature.GeneWindow
  , umms.core.feature.GenomeWindow
  , umms.core.feature.Window
- addState()
  : broad.core.hmm.MarkovModel< T >
- addStringArg()
  : broad.core.parser.CommandLineParser
- addStringListArg()
  : broad.core.parser.CommandLineParser
- addSuffixToName()
  : broad.pda.gene.GeneWithIsoforms
  , umms.core.annotation.Gene
- addToCentroid()
  : broad.core.motif.PositionWeightMatrix
- addToCluster()
  : broad.pda.geneexpression.clustering.Cluster
- addTranscript()
  : broad.core.primer3.HairpinKmer
- addVertex()
  : broad.pda.seq.graph.ChromosomeWithBubblesJGraphT
  , umms.core.scripture.OrientedChromosomeTranscriptGraph
- addWindow()
  : broad.core.siphy.TreeScalerIO
- AgilentArrayStats()
  : broad.pda.geneexpression.agilent.AgilentArrayStats
- AgilentProbe()
  : broad.pda.geneexpression.agilent.AgilentProbe
- AgilentToGCT()
  : broad.pda.geneexpression.agilent.AgilentToGCT
- align()
  : jaligner.NeedlemanWunsch
  , jaligner.NeedlemanWunschGotoh
  , jaligner.SmithWatermanGotoh
  , umms.core.alignment.SmithWatermanAlignment
- Alignment()
  : jaligner.Alignment
- AlignmentCount()
  : umms.core.model.AlignmentModel.AlignmentCount
  , umms.core.model.JCSAlignmentModel.AlignmentCount
- AlignmentDataModelStats()
  : broad.pda.seq.segmentation.AlignmentDataModelStats
- AlignmentModel()
  : umms.core.model.AlignmentModel
- Alignments()
  : broad.pda.datastructures.Alignments
- AlignmentsReader()
  : broad.pda.datastructures.AlignmentsReader
- allIsoformsToBED()
  : broad.pda.gene.GeneWithIsoforms
- almostContains()
  : broad.pda.gene.GeneWithIsoforms
  , umms.core.annotation.Gene
- almostContainsRefSeq()
  : umms.core.annotation.Gene
- almostEqual()
  : broad.core.annotation.BasicLightweightAnnotation
  , broad.core.annotation.LightweightGenomicAnnotation
  , broad.core.sequence.SequenceRegion
  , umms.core.annotation.Gene
- almostSameStructure()
  : umms.core.annotation.Gene
- alwaysFalse()
  : umms.core.general.Predicates
- alwaysTrue()
  : umms.core.general.Predicates
- AMEResultReader()
  : broad.core.motif.meme.AMEResultReader
- and()
  : umms.core.general.Predicates
- annotation()
  : broad.core.annotation.AnnotationHandler
  , broad.pda.datastructures.LocationAwareMatrix
- AnnotationList()
  : umms.core.annotation.AnnotationList< T extends Annotation >
- AnnotationOutOfBoundsException()
  : umms.core.coordinatesystem.AnnotationOutOfBoundsException
- AnnotationReader()
  : broad.core.annotation.AnnotationReader< T extends GenomicAnnotation >
- ANOVA()
  : broad.core.math.ANOVA
- anovaFStat()
  : broad.core.math.Statistics
- append()
  : broad.core.datastructures.MatrixWithHeaders
  , broad.core.multiplealignment.MAFAlignment
  , broad.core.multiplealignment.MultipleAlignment
  , broad.core.sequence.FastaSequenceIO
  , broad.core.sequence.Sequence
- appendColumns()
  : broad.core.datastructures.MatrixWithHeaders
- appendRows()
  : broad.core.datastructures.MatrixWithHeaders
  , broad.pda.datastructures.LocationAwareMatrix
- areRepeatsInDeepDirHierarchy()
  : broad.pda.feature.genome.DirectoryInstalledGenomeAssembly
- array2List()
  : umms.esat.ESAT
  , umms.esat.ESATDistanceCalculator
- arrayLeftDivide()
  : Jama.Matrix
- arrayLeftDivideEquals()
  : Jama.Matrix
- arrayRightDivide()
  : Jama.Matrix
- arrayRightDivideEquals()
  : Jama.Matrix
- arrayTimes()
  : Jama.Matrix
- arrayTimesEquals()
  : Jama.Matrix
- asAnnotation()
  : umms.core.alignment.AbstractPairedEndAlignment
- asDouble()
  : broad.core.parser.StringParser
- asFloat()
  : broad.core.parser.StringParser
- asInt()
  : broad.core.parser.StringParser
- asLong()
  : broad.core.parser.StringParser
- asRPKM()
  : umms.core.model.score.CountScore
- asString()
  : broad.core.parser.StringParser
- average()
  : broad.core.math.Statistics
- AverageBranchLength()
  : broad.core.siphy.tools.conservation.AverageBranchLength


---

Generated on Fri Aug 15 2014 15:11:16 for ESAT by  

 1.8.7
